# Preparation and Properties of Jute Fiber Long-Chain Fatty Acid Esters in Supercritical Carbon Dioxide

**DOI:** 10.3390/ma12091499

**Published:** 2019-05-08

**Authors:** Chong Li, Yueping Jiang, Baoshan Huang, Menghang Zhang, Yanhong Feng, Zhitao Yang

**Affiliations:** 1 State Key Laboratory of Pulp and Paper Engineering, South China University of Technology, Guangzhou 510640, China; lcly3278@126.com (C.L.); jyp19880905@163.com (Y.J.); dream_possible@126.com (M.Z.); 2 National Engineering Research Center of Novel Equipment for Polymer Processing, South China University of Technology, Guangzhou 510640, China; 3 Key Laboratory of Polymer Processing Engineering, Ministry of Education, South China University of Technology, Guangzhou 510640, China; 4 School of Materials and Environment, Beijing Institute of Technology, Zhuhai 519085, Guangdong, China; 5 School of Industrial Automation, Beijing Institute of Technology, Zhuhai, 519085, Guangdong, China; jetson_sbs@163.com

**Keywords:** continuous screw-extrusion steam explosion, supercritical carbon dioxide, jute fiber, plasticization, hydrophobicity

## Abstract

A two-step method involving continuous screw-extrusion steam explosion (CSESE) pretreatment and esterification in supercritical carbon dioxide (scCO_2_) is used to prepare long-chain fatty acid-modified jute fiber. The weight gain percentage (WG %) of CSESE-pretreated jute laurate (JL) was 110.7% when esterification was carried out in scCO_2_ at 14 MPa and 100 °C for 2 h. The corresponding WG % was 105.5% when esterification was instead carried out in pyridine at 100 °C for 2 h. Scanning electron microscopy and X-ray diffraction indicated that CSESE pretreatment enhanced the reactivity of jute fiber, with esterification in scCO_2_ simultaneously occurring on the fibers surface and internal walls. The glass transition temperature of esterified jute was approximately 119 °C, indicating that it could be hot processed over a wide temperature range. The esterified jute had an oil absorption ratio of 17.01 g/g, so it can be used as an oil absorption material.

## 1. Introduction

Cellulose is composed of β(1→4) linked D-glucose units, and is one of the most abundant natural polymers on earth. Cellulose is an important raw material and has many uses, such as in textiles, papers, foods, cosmetics, and biomaterials [1,2]. Hydroxyl groups of cellulose form strong intermolecular and intramolecular hydrogen bonds, and van der Waals interactions form a resistant microfibril network that gives cellulose its natural strength and reactivity [3].

Cellulose has strong hydrogen bonding and a high degree of crystallinity, so it is neither meltable nor soluble in conventional organic solvents. These factors limit its application. Cellulose derivatives with tailored properties can be obtained by introducing different substituent groups. Chemical modification such as esterification [4,5,6,7,8] allows the broad application of cellulose derivatives. The esterification of cellulose includes homogeneous and heterogeneous esterification. Solvents are essential for the homogeneous esterification of cellulose. A limited number of solvent systems have shown promise as cellulose solvents, including *N*,*N*′-dimethylacetamide/lithium chloride (DMAc/LiCl), 4-methylmorpholine-N-oxide (NMMO), 1-allyl-3-methylimidazolium chloride [Amim]Cl, tetrabutylammonium fluoride/dimethyl sulfoxide (TBAF/DMSO), and sodium hydroxide/urea [7,9,10,11]. These solvent systems have many obvious shortcomings, such as their poor environmental-friendliness, complicated and time-consuming preparation involving multiple steps, toxicity, and their low solubility of cellulose [12,13,14]. The heterogeneous esterification of cellulose also has drawbacks, such as the low degree of substitution and poor control over the reaction [15]. A more efficient, controllable, and environmentally-friendly method for cellulose esterification is therefore desirable.

Lignocellulose is one of the main sources of cellulose. The main components of lignocellulose are cellulose, lignin, hemicellulose, etc. Different lignocellulosic materials have different percentages of cellulose, lignin, and hemicellulose due to their species and origin. In lignocellulose, the three major components are strongly bonded by chemical bonds or non-covalent bonds, which makes it difficult to separate, transform, and modify lignocellulose. Jute fiber is a kind of lignocellulose with high cellulose content, and is naturally resistant to biological and chemical decomposition [16,17,18,19]. It is necessary to pretreat lignocellulose before use to improve its availability to reagents and therefore reactivity. Pretreatment methods for lignocellulose can be categorized as physical, physic-chemical, chemical, biological, or any combination of these [20]. Steam explosion (SE) pretreatment is a physical-chemical method that is efficient and non-polluting, so has attracted much attention. However, most steam explosions are batch processes with a short blasting time and thus are unsuitable for industrialization, have low efficiency, high energy consumption, etc. [21]. So, developing improved continuous steam explosion processes is important for the effective pretreatment of lignocellulosic materials. Continuous screw-extrusion steam explosion (CSESE), our laboratory-designed experimental device, has low energy consumption, no pollution, suitable for industrialization, etc., and is a continuous high-efficiency pretreatment method that dramatically decreases the fiber size, which improves accessibility and subsequent reactivity [22]. Moreover, the intensity of the CSESE pretreatment process could be adjusted by increasing/decreasing the number of pretreatment cycles for different biomass feedstocks, depending on application requirements, and the output reached 100–150 kg/h. And our research group has used this method to pretreat eulaliopsis binata, eucalyptus, sisal, sugar beet pulp, bagasse, corn cob, etc., and the CSESE process was feasible. Therefore, it is useful to pretreat jute fiber by CSESE before it is chemically modified.

An environmentally-friendly medium is also desirable for carrying out chemical reactions. Carbon dioxide is the most commonly used supercritical fluid due to its moderate critical constants (Tc = 31.1 °C, Pc = 7.38 MPa), nonflammability, nontoxicity, low cost, and chemical inertness. The removal of supercritical CO_2_ (scCO_2_) after carrying out the chemical transformation is not energy consuming, and the scCO_2_ can be readily recycled. These attributes make scCO_2_ an environmentally-friendly replacement for organic solvents in many applications, especially chemical reactions [23,24,25]. Various studies have reported the esterification of biomass in scCO_2_, such as the acetylation of cellulose [26], synthesis of cellulose carbamate [25,27], and esterification of starch [28,29]. However, the esterification of jute fiber treated by CSESE in scCO_2_ has not been reported.

Recent high-profile oil spills have resulted in widespread attention about water pollution, so developing oil absorption materials has important practical significance. Current oil absorption materials are mostly non-degradable [30,31,32]. The esterification of jute fiber treated by CSESE is an effective way to improve its oil absorption performance, and the esterification products have better biodegradability than synthetic polymer based oil absorption material.

Cellulose is also difficult to plasticize and process, because of its high crystallinity and melting temperature. Esterification is an effective method for improving the plasticization of cellulose. The plastic modification of cellulose has mainly been carried out by homogeneous esterification [33,34,35]. Cellulose laurate was prepared in LiCl/DMAc and its tensile strength was reportedly 4.84 MPa [36]. There have been very few such reports exploiting heterogenous esterification, and the mechanical properties of cellulose esterification products have rarely been reported.

In the current study, long-chain fatty acid-modified jute fiber was prepared in a two-step process involving CSESE pretreatment and subsequent esterification in scCO_2_. Specifically, jute fiber was treated by CSESE, then reacted with lauroyl chloride in scCO_2_. The structure and morphology of the obtained products were analyzed by Fourier-transform infrared (FTIR) spectroscopy, solid-state ^13^C nuclear magnetic resonance (^13^C NMR) spectroscopy, and scanning electron microscopy (SEM). The degree of reaction was then investigated by varying the scCO_2_ pressure and the reaction temperature and time. The obtained products were investigated using contact angle (CA) measurements, X-ray diffraction (XRD), thermogravimetric analysis (TGA), mechanical property measurements, and dynamic thermo-mechanical analysis (DMA). Finally, the plasticization and oil absorption properties of the esterification products were investigated.

## 2. Materials and Methods 

### 2.1. Materials

Jute fiber was obtained from Nanjing Xinhe Textile Co., Ltd. (Nanjing, China). Prior to use, jute fiber was pretreated by CSESE as described previously [22]. Briefly, Jute fiber was cut into short fibers with lengths of approximately 10 mm. The screw continuously conveyed the jute fiber chips with about 50% moisture content forward, where they were squashed and compacted by the screw, and gradually heated due to friction between the chips, screw, and barrel. The pressure and temperature of the compacted jute fiber reached approximately 1–1.5 MPa and 120–150 °C, respectively, the screw speed was about 300 rpm, when they conveyed to the die. The compacted jute fiber was continuously discharged from the die, with a slit width of 1 mm. Pressurized water in the fiber bundles was instantaneously vaporized, which resulted in destruction of the tissue structure of the fiber bundles. This pretreatment process was repeated three times to obtain pretreated jute fiber, and the output reached 100–150 kg/h. Then the pretreated jute fiber was washed with tap water and dry naturally [37,38], CSESE pretreated jute fiber is hereafter referred to as JSE. Analytical grade lauroyl chloride, pyridine, and anhydrous ethanol were purchased from Aladdin (Shanghai, China), Sinopharm Chemical Reagent Co., Ltd. (Shanghai, China), and Nanjing Chemical Reagent Co., Ltd. (Nanjing, China), respectively. High purity CO_2_ (≥99.9% by volume) was used. All chemicals were used without further purification.

### 2.2. Reactor Setup

**Figure 1:**
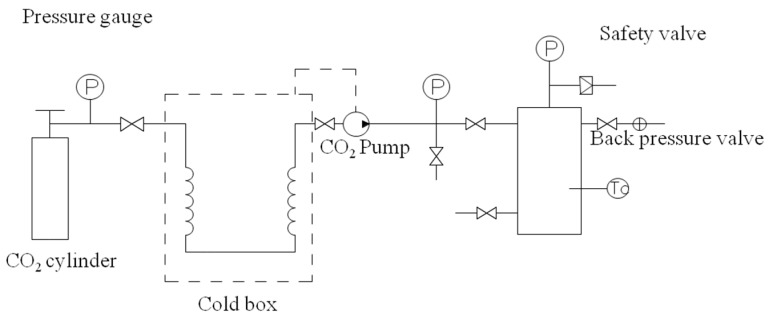
The schematic diagram of the supercritical carbon dioxide (scCO_2_) device.

The reactor was purchased from Haian Oil Scientific Research Apparatus Co., Ltd. (Nantong, China). The high pressure setup includes a carbon dioxide cylinder, external circulation refrigeration unit, high pressure pump, temperature control unit, and a high temperature autoclave reactor with a volume of 300 mL (Figure 1). The reactor is equipped with an overhead stirrer. The high pressure pump unit consists of a membrane pump with a capacity of 6 L/h at a maximum pressure of 40 MPa. To prevent cavitation in the pump, CO_2_ is first cooled to 0 °C in a heat exchanger attached to the external circulation refrigeration unit. The temperature control unit is then used to heat the CO_2_ to the desired temperature.

### 2.3. Experimental Procedure

#### 2.3.1. Esterification of JSE in scCO_2_

**Figure 2:**
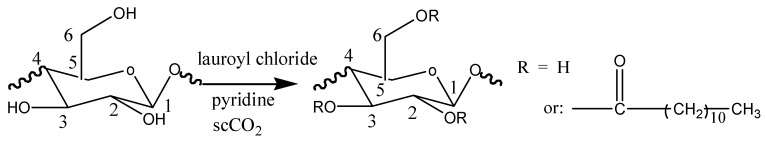
Synthesis of continuous screw-extrusion steam explosion (CSESE) pretreated jute fiber (JSE) laurate in scCO_2_.

Dried JSE (2.0 g), pyridine (2.25 mol/mol of anhydroglucose units (AGU)) and lauroyl chloride (3 mol/mol of AGU) were stirred in a 100 mL polytetrafluoroethylene-lined autoclave, which was then transferred into the CO_2_ reactor. The autoclave was then sealed and heated to the desired temperature. CO_2_ was added to increase the pressure to the desired level. The reaction contents were stirred at 200 r/min for a predetermined time. After reaction, the pressure was slowly decreased to standard atmospheric pressure, and the autoclave was naturally cooled to room temperature. The reaction mixture was then transferred to a beaker, and the product was precipitated with ethanol, collected by filtration, washed with ethanol, water, and ethanol again, followed by drying at 60 °C in an oven. The obtained product was JSE laurate and is hereafter abbreviated as JL. The synthesis of JL in scCO_2_ is shown in Figure 2.

#### 2.3.2. Esterification of Jute Fiber in scCO_2_

The esterification of jute fiber in scCO_2_ was carried out similarly to the esterification of JSE, at 14 MPa and 100 °C for 2 h. The esterification product is hereafter abbreviated as R-JL.

#### 2.3.3. Esterification of JSE in Pyridine

Dried JSE (2.0 g), pyridine (30 mL), and lauroyl chloride (3 mol/mol of AGU) were placed in a 250 mL round bottomed flask with attached reflux condensation and agitation. The reaction was carried out at 100 °C for 2 h. Afterwards, the reaction mixture was transferred to a beaker, and the product was precipitated with ethanol, collected by filtration, washed with ethanol, water, and ethanol again, followed by drying at 60 °C in an oven. The obtained product is hereafter abbreviated as JL-Py.

### 2.4. Characterization

#### 2.4.1. FTIR Spectroscopy

Dried JSE or JL was cut into small particles, then mixed with dried potassium bromide powder in an agate mortar. The mixture was ground to a fine powder, which was pressed into a disc and then dried for 30 min in an infrared box. The dried discs prepared from JSE and JL were then used to collect FTIR spectra using a Nicolet-Nexus 670 infrared spectrophotometer (Thermo Nicolet Corporation, Madison, WI, USA).

#### 2.4.2. ^13^C NMR Spectroscopy

^13^C (CP/MAS) NMR spectra of samples were recorded using a superconducting Fourier- transform nuclear magnetic resonance spectrometer (AVANCE III HD 400 spectrometer, Bruker, Karlsruhe, Germany) in dual resonance MAS Mode, with a standard 4-mm-diameter probe. Chemical shifts were referenced to the signal of tetramethylsilane (TMS, 0 ppm).

#### 2.4.3. SEM

Images of the morphologies of JSE and JL were obtained using a scanning electron microscope (model FEI Quanta FEG 250, Hillsboro, OR, USA) operated in secondary electron mode, with a beam current of 100 mA and an accelerating voltage of 20 kV. Prior to analysis, samples were coated with a thin layer of gold using as a vacuum coater to prevent charging.

#### 2.4.4. XRD

XRD patterns of JSE and JL were obtained using a Bruker D8 ADVANCE (Bruker) diffractometer (40 kV, 40 mA) by the refraction method, using nickel-filtered Cu Kα radiation (λ = 1.54 Å). Scans were performed from 4° to 60° 2θ in increments of 0.04° and a scan rate of 0.2 s per step. The collected data were analyzed using MDI Jade (6.0, Materials Data Incorporated, Berkeley, CA, USA). The peaks of crystalline and amorphous fractions were obtained by means of XRD-peak- different-imitating analysis with parameters for all samples. The error in the data fitting was approximately 3%.

#### 2.4.5. TGA

The thermal stabilities of JSE and JL were studied using a thermogravimetric analyzer (TG209 F3, Netzsch, Selb, Germany). Approximately 6 mg of sample was heated from 30 to 700 °C at a heating rate of 10 °C/min. Nitrogen gas at a flow rate of 50 ml/min was used to protect samples from oxidation.

#### 2.4.6. Static Water CA Measurements

CA values of 3 μL water droplets on the molded films were measured using an automatic video microcontact angle measuring instrument (DATA physics, DCa40 MICRO, Stuttgart, Germany).

#### 2.4.7. Mechanical Properties

The mechanical properties of the molded samples were measured using a universal material testing machine (Instron 5566, Boston, MA, USA) at room temperature. The specific test procedure is according to the literature [35].

#### 2.4.8. Dynamic Thermomechanical Analysis (DMA)

A dried sample was ground to a powder, which was pressed into a slice with a diameter and thickness of approximately 15 mm and 2 mm, respectively, using a purpose-built molding apparatus (TA Instruments, Wilmington, DE, USA). The resulting disc was then subjected to testing in compression mode, using a frequency of 10 Hz, amplitude of 10 μm, heating rate of 3 °C/min, and temperature range of −80 °C to 180 °C.

#### 2.4.9. Oil Absorption Performance

Dried JSE or JL (0.1 g) was accurately weighed onto a stainless-steel wire mesh, which was then immersed in 250 mL of soybean oil. After 15 min, oil was removed and it is allowed to drain for 5 min. The sample was then weighed, and the oil absorption ratio was calculated from [39]:

Q = (m_w_ − m_0_)/m_0_, (1)

where Q is the oil absorption ratio, and m_0_ and m_w_ are the weight of the material before and after oil absorption, respectively.

### 2.5. Determination of Weight Gain Percentage

The weight gain percentage (WG %) was calculated according to Equation (2) [4]:

WG % = (m_1_ − m_0_)/m_0_ × 100%, (2)

where m_0_ is the weight of JSE, and m_1_ is the weight of JSE after esterification (i.e., the mass of JL).

The cellulose content of jute is high, so cellulose can be used to estimate the degree of substitution of lauroyl chloride. The molecular weight of C_6_H_10_O_5_ (i.e., the AGU) on the macromolecule chain of cellulose is 162 g/mol. The molecular weight of the lauroyl chloride is 218.5 g/mol. The hydrogen of a free hydroxyl group on the AGU is substituted by a lauroyl group. After esterification, the unit has a molecular weight of 182 g/mol. The WG % of JL is therefore 182/162, so the relationship between the degree of substitution (DS) and the WG % of JSE can be estimated. The DS was therefore estimated according to Equation (3):

DS = WG %/(182/162).(3)

## 3. Results and Discussion

### 3.1. Esterification of JSE and Jute Fiber

**Figure 3:**
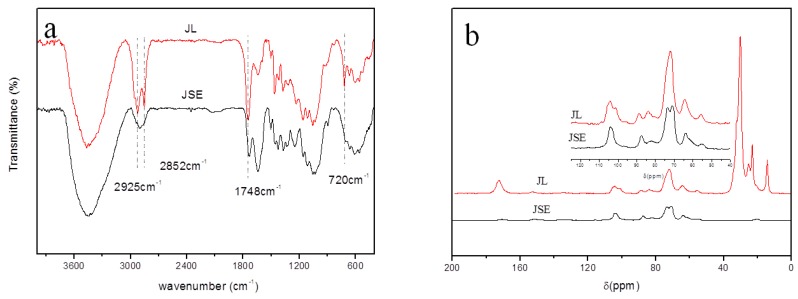
FTIR (**a**) and ^13^C NMR (**b**) spectra of pretreated jute fibers (JSE) and JSE laurate (JL).

FTIR spectra of JSE and JL are shown in Figure 3a. The spectrum of JSE has an absorption band at 2901 cm^−1^, and the spectrum of JL has two additional absorption bands at 2925 and 2852 cm^−1^ (antisymmetric and symmetric stretching vibrations of –CH_2_– and –CH_3_ groups), which collectively indicate the presence of long aliphatic chains. Compared to JSE, two new bands are observed in the spectrum of JL. The first at 1748 cm^−1^ (–C=O stretching vibration) [2] corresponds to the vibration of carbonyl ester groups. The second at 720 cm^−1^ is attributed to four or more linearly connected –CH_2_– groups (–(CH_2_)_4_-rocking vibration) [36]. This indicates that fatty substituents have been linked directly to cellulose in JSE. The absence of absorptions at 1800 cm^−1^ indicates the absence of free lauroyl chloride in JL [4]. In summary, these spectra are consistent with JL being synthesized from JSE and lauroyl chloride.

^13^C NMR spectra of JSE and JL are shown in Figure 3b. The synthesis of JL in scCO_2_ is shown in Figure 2. Signals at 50–110 ppm are predominantly attributed to the different carbons of cellulose in JSE and JL [2,5,6,40]. Signals in the spectrum of JSE are assigned as follows: 104.1 ppm (C-1), 87.6 ppm (C-4 crystalline), 82.3 ppm (C-4 amorphous), 73.6 ppm (C-5, C-3), 71.1 ppm (C-2), and 63.9 ppm (C-6) in the AGU structure. Signals in the spectrum of JL are assigned as follows: 104.1 ppm (C-1 crystalline), 101.3 ppm (C-1 amorphous), 88.8 ppm (C-4 crystalline), 83.9 ppm (C-4 amorphous), 82.5 ppm (C-4 amorphous), 72.1 ppm (C-5, C-3, C-2), and 64.3 ppm (C-6). The signal at 55.6 ppm is assigned to the methoxyl peak of lignin [4]. Compared with spectrum of JSE, new peaks at 14.4, 23.2, 25.2, 30.2, 101.3, and 172.4 ppm are present in the spectrum of JL. These are attributed to the –CH_3_ group, –CH_2_– groups (20–40 ppm), C-1 (amorphous), and ester carbonyl carbon of the lauroyl group introduced into JSE [7]. The signals of the incorporated aliphatic carbons occur at high magnetic fields and are sufficiently separated from those of saccharide moieties. Therefore, the ^13^CNMR results are consistent with lauroyl groups introduced into JSE chains through reaction with the hydroxyl groups of cellulose. Compared with the spectrum of JSE, the increased intensities of cellulose peaks at 101.3, 83.9, and 72.1 ppm indicate that the crystalline region of cellulose is partially destroyed and that it participates in the esterification reaction. As a result of this, the proportion of the amorphous fraction increases. These results are consistent with esterification between cellulose and lauroyl chloride in scCO_2_, and the introduction of acyl groups into cellulose.

### 3.2. Optimization of Esterification Conditions

**Figure 4:**
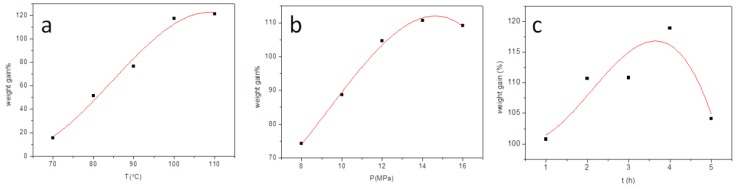
Change in WG % with reaction temperature (**a**), scCO_2_ pressure (**b**), and reaction time (**c**). In (**a**), the scCO_2_ pressure is 8 MPa and the reaction time is 2 h. In (**b**), the reaction time is 2 h and the reaction temperature is 100 °C. In (**c**), the scCO_2_ pressure is 14 MPa and the reaction temperature is 100 °C.

scCO_2_ is highly compressible and its solvent properties can be tuned over a wide range by varying the pressure and temperature [27]. Varying the scCO_2_ pressure, reaction temperature, and duration affects the ability of reactants to penetrate the fiber. Reactions were carried out at varying scCO_2_ pressures and reaction temperatures and durations, to optimize the reaction conditions (Figure 4). The influence of the reaction temperature on the WG % was first investigated from 70 to 110 °C. Figure 4a shows that the WG % increases with increasing temperature, which reflects the increasing reaction rate. The higher temperature promotes the esterification reaction, so enhances the degree of esterification of cellulose in JSE. Thus, the WG % increases with increasing reaction temperature. When the reaction temperature is >100 °C, a black product is obtained as a result of degradation of the JSE matrix and its derivatives. At high temperature, HCl bound to pyridine can be released, and the presence of HCl also accelerates the degradation of the JSE matrix and its derivatives. An esterification temperature of 100 °C is therefore considered appropriate.

The effect of scCO_2_ pressure on the WG % is shown in Figure 4b. The WG % first increases from 74.3% to 110.7%, reaching a maximum at a pressure of 14 MPa. The WG % slightly decreases with further increase in scCO_2_ pressure. This is related to many factors such as the solubility of lauroyl chloride and pyridine in scCO_2_, the swelling effect and compressive effect of scCO_2_, the capability of scCO_2_ as a carrying agent, or a combination of several competing factors [25,27,41,42]. At low pressure, the solvent intensity of CO_2_ is insufficient to swell the JSE substrate enough to permit the rapid penetration of lauroyl chloride and pyridine. At high pressure, scCO_2_ is a much better solvent and carrying agent for lauroyl chloride and pyridine. The swelling effect of scCO_2_ on the JSE matrix can enhance the diffusion rate of the reaction reagent into JSE, leading to higher WG %. The most favorable combination of these factors for the esterification of JSE is reached at 14 MPa. The WG % decreases with further increase of scCO_2_ pressure. This may reflect a lower free volume in the JSE matrix due to compression, which would reduce the diffusion of reactants into JSE, lower the reaction rate, and lead to a lower WG %. Another factor could be that JL is more prone to degradation in the presence of a small amount of free HCl at higher pressure, which would also decrease the WG %. At high pressure, a fraction of the extracts and lignin from the JSE matrix can be extracted by scCO_2_ and lost during pressure relief, which would also decrease the WG %. Therefore, an scCO_2_ pressure of 14 MPa is considered optimum and is used for subsequent experiments.

The effect of reaction time on the WG % was then studied. Figure 4c shows that the WG % also increases with increasing esterification time. A longer time promotes the esterification reaction and enhances the degree of esterification for cellulose in JSE. A maximum WG % occurs when the esterification time is approximately 4 h, and the WG % decreases with further increase in reaction time. This could be because of the partial pyrolysis of components of the JSE matrix (i.e., hemicellulose and extracts) and its derivatives. An esterification time of 4 h is therefore considered suitable.

A maximum WG % of 123.5% is obtained at an optimum reaction temperature of 100 °C, scCO_2_ pressure of 14 MPa, and reaction duration of 4 h. The DS is estimated to be 1.10 according to Equation (3). This is higher than reported values [43,44,45] in the heterogeneous esterification of long chain fatty esters of plant fibers. The degree of esterification between JSE and lauroyl chloride can be readily controlled by changing the temperature and/or scCO_2_ pressure.

### 3.3. Contrast Experiment

For comparison, the reaction of JSE with lauroyl chloride in pyridine was carried out at a temperature of 100 °C and reaction time of 2 h. The WG % of JL-Py is 105.5%. The WG % of JL in scCO_2_ (100 °C, 2 h) is higher at 110.7%. This is because scCO_2_ acts as a swelling agent for JSE and carrying agent for lauroyl chloride and pyridine, which promotes the esterification reaction.

The reaction of jute fiber with lauroyl chloride was then carried out in scCO_2_ (100 °C, 14 MPa, 2 h), and the resulting WG % of R-JL is 93.5%. The higher WG % of JL is attributed to CSESE pretreatment providing a smaller fiber diameter and larger specific surface area, both of which promote the esterification reaction.

### 3.4. Morphological Structure

**Figure 5:**
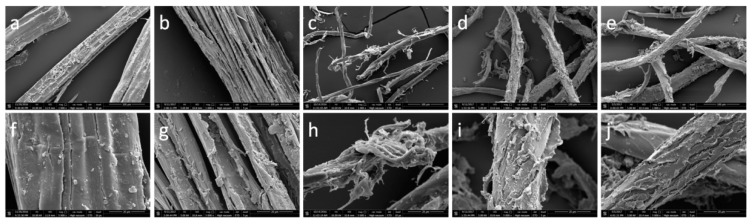
SEM images of jute (**a**,**f**), Jute laurate (R-JL, **b**,**g**), pretreated jute (JSE, **c**,**h**), JSE laurate prepared in Pyridine (JL-Py, **d**,**i**), and JSE laurate (JL, **e**,**j**).

The microstructures of jute and R-JL are shown in Figure 5. Figure 5a,f shows that jute has a fibrous structure with a relatively smooth surface and protrusions created by cell cavities. The surface contains debris left during the preparation of the jute fiber. The surface of R-JL is significantly different, as shown in Figure 5b,g. Fiber bundles of R-JL undergo significant splitting, and bundles are destroyed by the swelling of scCO_2_ and the rapid release of pressure after reaction. While separation is observed in the intercellular layer, there is no obvious damage to the cell wall, so the cell diameter does not change significantly. Therefore, the esterification product is produced mainly on the fiber surface.

Figure 5c,h shows that the surface of JSE is rough and partly fractured as a result of CSESE pretreatment [22,38,46]. CSESE damages and fractures the fiber bundles of jute fiber, generating small irregular particles with rough and partly fractured surfaces.

The surface morphologies of JL-Py in Figure 5d,i and JL in Figure 5e,j differ significantly from that of JSE. The surfaces of JL-Py and JL are rougher and are covered with a layer of material which could be products of the reaction between JSE hydroxyl groups and lauroyl chloride. The surface layer material of JL appears denser than that of JL-Py, indicating that the degree of esterification of JSE in scCO_2_ is greater than that in pyridine. This is consistent with the result of the above contrast experiment. The average fiber diameter of JL is much larger than those of JL-Py and JSE. This could be due to the swelling effect of scCO_2_ and the introduction of acyl substituents onto the internal cell wall of cellulose. Thus, hydroxyl groups on both the JSE surface and internal wall are proposed to be involved in the esterification reaction with lauroyl chloride. This leads to the high DS (1.10). The swelling of JSE in scCO_2_ and esterification of the surface and interior hydroxyl groups of JSE also disrupts partially crystalline regions and forms amorphous regions. This conclusion is consistent with the ^13^CNMR results showing that crystalline regions of cellulose are partially destroyed and participate in esterification. Compared with jute, JSE has a relatively small fiber diameter, a large specific surface area, and higher accessibility, so the WG % of JL is higher than that of R-JL.

The surface morphologies of R-JL (Figure 5b,g) and JL (Figure 5e,j) also significantly differ. The surface of fiber bundles in R-JL is covered with reaction products. JL has esterification products on the surface and internal cell walls. This indicates that the degree of esterification in JSE should be higher than that in jute, i.e., the WG % of JL is higher than that of R-JL. This is because the internal cell walls in JL are also involved in esterification, so the diameter of fiber bundles in JL is larger. This is consistent with the above control experiment results.

### 3.5. Crystallization Properties

**Figure 6:**
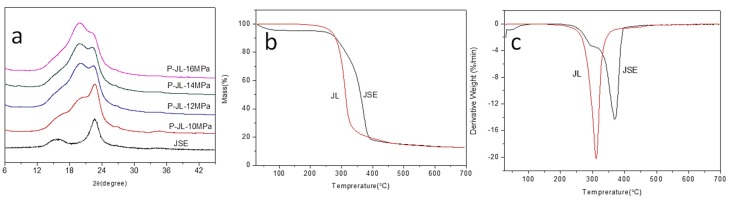
X-ray diffraction (XRD) patterns (**a**), thermogravimetric (TG) curves (**b**), and differential thermogravimetric (DTG) curves (**c**) of pretreated jute (JSE) and JSE laurate (JL).

XRD patterns of JSE and JL are shown in Figure 6a. JSE exhibits the typical diffraction pattern of cellulose type I, with the main diffraction peaks at 2θ = 16.0°, 22.6°, and 34.6° [30]. The XRD pattern of JL differs to that of JSE. The former contains an additional peak at 2θ = 19.86°, which is due to ordering of the long-chain fatty acyl groups of the cellulose chains [2,47]. This result is consistent with the conclusion that JSE is esterified with lauroyl chloride. The relative weakening of cellulose diffraction peaks (2θ = 22.6°) after esterification is attributed to the reduction of hydroxyl groups. This is because crystalline regions of cellulose are partially destroyed and participate in esterification. This result is consistent with the above ^13^CNMR result. The intensity of the 2θ = 19.86° peak increases with increasing scCO_2_ pressure. This demonstrates that the degree of reaction increases with increasing scCO_2_ pressure, so the amount of hydroxyl groups on cellulose decreases, while the amount of long chain fatty ester groups on cellulose increases. This is because the degree of esterification increases with increasing scCO_2_ pressure, i.e., the degree of substitution of lauroyl groups increases. However, the WG % of JL decreases with increasing scCO_2_ pressure. This may be due to a reduction in free volume, and/or the degradation of JL caused by a small amount of free HCl, and/or a fraction of extracts and lignin from the JSE matrix being extracted by scCO_2_ at high pressure (16MPa).

### 3.6. Thermal Properties

Substituents have a greater impact on the thermal stability of cellulose and its derivatives [48]. Therefore, the thermostabilities of JSE and JL were investigated using thermogravimetric (TG) and differential thermogravimetric (DTG) analyses. The results are shown in Figure 6b and c, respectively. The initial decomposition temperatures of JSE and JL are 355.2 and 289.8 °C, respectively. The temperatures at the maximum rate of weight loss for JSE and JL are 370.1 °C and 311.3 °C, respectively. These results show that the thermal stability of JL is lower than that of JSE. This is attributed to the introduction of less stable side chains (lauroyl groups). It is also attributed to the reduction of hydroxyl groups, which leads to disruption of intermolecular and intramolecular interactions such as hydrogen bonds of JSE chains. Crystalline regions of cellulose are partially destroyed during esterification, so JL with a looser and more disordered structure is more easily thermally decomposed. The decomposition temperature range of JL is also narrower than that of JSE. This may be caused by two reasons. The first is the "inducing" effect of the lauroyl groups. Lauroyl groups have a lower thermal stability than hydroxyl groups. Introducing lauroyl groups into the molecular chain of cellulose induces the degradation of cellulose. The temperature of the maximum thermal degradation rate decreases, and the temperature range of thermal degradation becomes narrower. The second reason is that hydroxyl groups (–O–H) of cellulose chains are replaced by ester bonds (–O–C) during the esterification of JSE. The –O–H bond energy (463 kJ/mol) is greater than that of –O–C (326 kJ/mol). Therefore, the thermal stability decreases during esterification, and the temperature range of the thermal degradation of JL is narrower than that of JSE.

### 3.7. Hydrophobic Properties

**Figure 7:**
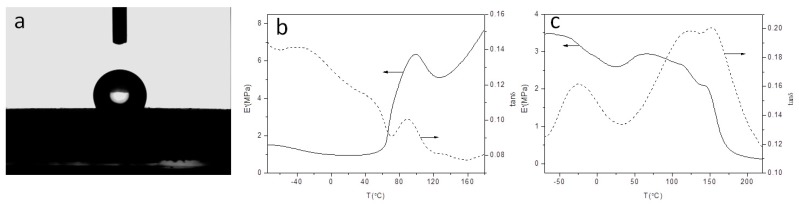
Optical image of a water droplet on JL-14 showing hydrophobicity (**a**), dynamic thermo-mechanical analysis (DMA) curves of pretreated jute (JSE, **b**), and DMA curves of JSE laurate (JL, **c**).

CA measurements were carried out for JSE and JL to evaluate their hydrophobicity. Water droplets spread and are quickly absorbed by JSE, so the CA is difficult to measure. This occurs because the individual fibers are not tightly integrated, and the water droplet readily penetrates into the fiber which indicates that JSE is highly hydrophilic, and is expected because of the high hydroxyl group content of its cellulose fibers. Water droplets on JL samples prepared at various scCO_2_ pressures all have similar appearances. An optical image of a water droplet on JL is shown in Figure 7a, and CA values for specific samples are given in Table 1. The CA of JL increases with increasing scCO_2_ pressure, and all CA values are >90°. This indicates that JL is hydrophobic. The maximum CA is 118.1°, which is higher than values from reported studies [30,31]. There are two possible reasons for this. Hydrophobic lauroyl groups are introduced into the macromolecular chains of cellulose in JSE during esterification. Lignocellulose also significantly swells in scCO_2_, which increases the DS (up to 1.10) and increases the hydrophobicity of JL. The CA first increases and then decreases with increasing scCO_2_ pressure, reaching a maximum at 14 MPa (JL-14). This is because the degree of esterification first increases and then decreases with increasing pressure, and the degree of esterification is highest at 14 MPa. Lauroyl groups are hydrophobic, so the CA of JL-14 reaches its highest among the tested samples. This conclusion is consistent with the XRD results and effect of scCO_2_ pressure on the WG %.

### 3.8. DMA

Figure 7b,c shows the storage modulus (E′) and loss factor (tan δ) of JSE and JL as a function of temperature. It can be seen from the figure that as the temperature increases, the storage modulus (E′) of JSE slowly decreases, when the temperature reaches 60 °C, E′ begins to rise rapidly, and then E′ drops rapidly again, a loss tangent peak was observed at about 90 °C, and this mechanical state change behavior may be a glass transition caused by lignin [38]. Compared to JSE, the DMA curve of JL is significantly different. JL exhibits a first relaxation at below room temperature, as revealed by a first drop in E′ associated with a loss tangent peak. The first loss peak (tanδ1, –26 °C) is attributed to the alkyl side chain fraction not involved in the crystalline phase [36]. When the length of the side-chain alkyl group is greater than or equal to 12 carbon atoms, the side chain of the long-chain fatty ester of cellulose is partially crystalline [31]. When the temperature increases, a slow drop of E′ is observed accompanied by a broad loss tangent peak, which is caused by the overlap of the two loss peaks. The temperature of the two loss peaks is 119 °C and 151 °C, respectively. This mechanical relaxation behavior may be caused by the skeleton movement of the molecular chain and the "boat-chair" conformational structure of the glucose ring or the movement of the side chain oxycarbonyl group [28,29,33], that is, the change in mechanical state caused by modification of cellulose. The glass transition temperatures of cellulose, hemicellulose and lignin are 220–250 °C, 150–220 °C, and 130–205 °C [49], respectively. Compared with the three main components, JL has a lower mechanical relaxation temperature, and the storage modulus E′ of JL shows the characteristics of thermoplastic materials. This means that JL has certain thermoplastic characteristics, and the thermoplastic properties of JL is improved.

### 3.9. Mechanical Properties

JSE has a large amount of hydrogen bonding and high crystallinity, so is difficult to plasticize and process. JL has a tensile strength of 5.37 MPa and a fracture strain of 4.65%, indicating that it has better thermoplastic properties than JSE. The tensile strength of JL is higher than the reported previously [36]. This is because a large amount of lauroyl groups are grafted onto the cellulose chains in JSE. Lauroyl groups exhibit good thermoplasticity, so JL also exhibits relatively good thermoplasticity. The preparation of JL involves full-component jute fiber, so the JL matrix contains a certain amount of lignin and other components. The presence of lignin with its lower Tg promotes the plasticization of JL. Therefore, JL exhibits better thermoplasticity than JSE.

### 3.10. Oil Absorption Performance

To investigate the oil absorption performance before and after the esterification of JSE, the oil absorption ratios of JSE and JL were tested using soybean oil. The oil absorption ratios of JSE and JL are 8.42 and 17.01 g/g, respectively, which is explained by the higher surface roughness after esterification, as shown in Figure 5. After esterification, a large amount of laurate groups are introduced into JL, and the hydrophobicity of these groups increases the oil absorption ratio. The esterification of JSE also lowers the surface energy, which promotes the wetting of JL by the soybean oil. Therefore, the oil absorption ratio of JL is higher than that of JSE.

## 4. Conclusions

A two-step method involving CSESE pretreatment and subsequent esterification in scCO_2_ is used to prepare JL. Compared with esterification without CSESE pretreatment or esterification carried out in pyridine, the two-step method is favorable in modifying the jute fiber. CSESE pretreatment enhances the fiber reactivity, and scCO_2_ can then transport reagents to the fiber interior because of its high permeability. These factors lead to a high modification efficiency, with the degree of substitution reaching 1.1. JL prepared through the two-step method has a tensile strength of 5.37 MPa and a fracture strain of 4.65%, so JL has better thermoplastic properties. And the glass transition temperature of JL is 119 °C, indicating JL has a wide plasticizing processing window. In addition, JL has a better oil absorption ratio of 17.01 g/g and can be used as a biodegradable oil absorption material. Besides, the CSESE pretreatment is low energy consumption, no pollution, suitable for industrialization, and the esterification modification method is environmentally friendly, low energy consumption and high efficiency. Therefore, this method can be widely applied to plasticize and hydrophobicize of lignocellulose.

## Figures and Tables

**Table 1:** Water contact angles of the various samples.

**Sample**	JL-8	JL-10	JL-12	JL-14	JL-16
**Contact Angle (°)**	105.4	106.4	107.1	118.1	109.8

Note: JL: JSE laurate prepared in supercritical carbon dioxide, the number represents the pressure of scCO_2_.
